# Transforming healthcare delivery: a descriptive study of a novel provider-to-provider virtual care platform

**DOI:** 10.3389/fpubh.2023.1284566

**Published:** 2023-12-08

**Authors:** Karthik Tennankore, Jennifer Jones, Ashley Miller, Ashfaq Adib, Shan Mathew, Daniel Rasic, Jacob Cookey

**Affiliations:** ^1^Division of Nephrology, QEII - Dickson Building, Halifax, NS, Canada; ^2^Division of Digestive Care and Endoscopy, QEII - Victoria Building, Halifax, NS, Canada; ^3^Division of General Internal Medicine, QEII - Bethune Building, Halifax, NS, Canada; ^4^Virtual Hallway Consults Inc., Halifax, NS, Canada; ^5^Pleasant Street Medical Group, Dartmouth, NS, Canada

**Keywords:** virtual care solutions, Hallway medicine, healthcare solution, digital healthcare, peer to peer communications, healthcare technologies, synchronous communication technology, eConsult

## Abstract

**Introduction:**

Addressing challenges in access to specialty care, particularly long wait times and geographic disparities, is a pressing issue in the Canadian healthcare system. This study aimed to evaluate the impact and feasibility of provider-to-provider phone consultations between primary care providers (PCPs) and specialists using a novel virtual care platform in Nova Scotia (Virtual Hallway).

**Methods:**

We conducted a cross-sectional survey over 5 months, involving 211 PCPs and 34 specialists across Nova Scotia. The survey assessed the need for formal in-person referrals as well as clinician satisfaction. Statistical methods included descriptive statistics and the one-sample *t*-test.

**Results:**

We found that 84% of provider-to-provider phone consultations negated the need for an in-person specialist referral. It was also reported that 90% of patients that did require in-person consultation had enhanced care while they awaited an in-person appointment with a specialist. Very high levels of satisfaction were reported among both PCPs and specialists, and there was a noticeable increase in billing volumes related to these consultations as measured by provincial billing codes.

**Conclusion:**

The findings indicate that provider-to-provider phone consultations are feasible, well-accepted and also effective in reducing the need for in-person specialist visits. This approach offers a promising avenue for alleviating waitlist burdens, enhancing the quality of care, and improving the overall efficiency of healthcare delivery.

## Introduction

Access to specialty care remains a pivotal concern for healthcare systems around the world. This paper explores an innovative approach to enhancing this access through synchronous provider-to-provider communication between primary care providers and specialists, with a focus on the Canadian healthcare context. The central contribution of this study is to provide an empirical analysis of how digital platforms, specifically using a platform called Virtual Hallway, can streamline the referral process, reduce wait times, and democratize access to specialty healthcare services.

### Background

The Canadian healthcare system faces substantial hurdles in specialty care provision, with patients enduring lengthy wait times that span an average of 26 weeks, though this can vary between provinces (1). The crux of the issue lies not only in these protracted delays but also in the uneven distribution of healthcare specialists, who are predominantly located in urban areas. This geographic inequality necessitates often burdensome travel for rural inhabitants and disproportionately impacts those with limited resources (2). Adverse health and economic consequences are well-documented, with delays leading to severe outcomes such as increased morbidity and mortality (3, 4).

One potential solution to improve access to specialty care is to reduce the barriers to engaging in peer-to-peer consultation between primary care providers (PCPs) and specialists. Primary care physicians and nurse practitioners serve as the entry point for patients to access healthcare services, including specialty care. In most situations, a patient must be referred by a PCP to access specialty care. Referrals must be reviewed for appropriateness and completeness, triaged to determine urgency, and then patient appointments are subsequently booked based on information provided in a referral. However, it has been observed that many formal referrals and specialist-patient consultations could be avoided if the PCP could consult directly with the specialist, thereby reducing wait times and potentially improving patient outcomes (5).

Electronic consultations (eConsults) can facilitate PCPs seeking specialist advice digitally, in writing, and without the need for an in-person referral (6). Another promising avenue is the utilization of synchronous peer-to-peer consultations, whereby the PCP arranges a phone consultation with a specialist, receives verbal advice, and subsequently implements the recommended care plan. This approach has the potential to optimize access to specialty care and reduce waiting times for patients who do not require formal specialist consultation through specialist-PCP phone consults.

To date, the evidence for peer-to-peer communication in medical care has been limited by heterogeneity (i.e., differences in program type, outcome measures) as well as a paucity of studies specifically focusing on synchronous provider-to-provider phone consults (7).

### Purpose

This descriptive study sought to answer the following questions: how do primary care providers (PCPs) and specialists utilize Virtual Hallway (a novel phone consultation platform) for peer-to-peer communication? What impact does the platform have on in person referrals? What impact does it have on billing code usage? Is this novel virtual care platform satisfactory?

## Methods

### Setting

The pilot project was conducted in partnership with Nova Scotia Health and the Coordinated Accessible National (CAN) Health Network over a five-month period starting in May 2022. Nova Scotia has a population of just over 1 million people spread across approximately 55, 284 square kilometers. The participants involved nurse practitioners, family medicine specialists, and medical specialists. Physicians (both referring and consulting) have been eligible for compensation for synchronous provider-to-provider consultation since April 2017 as described in the Nova Scotia Medical Services Insurance Interim Fee Guide.^1^

### Virtual Hallway platform

Virtual Hallway is an online platform that facilitates provider-to-provider patient-focused virtual consultation via synchronous telephone conversations. To initiate a phone consult request, a requesting provider (usually a primary care provider but occasionally a specialist) logs onto the Virtual Hallway system and completes an electronic form for a patient-specific question, with an option to attach any relevant patient documents (e.g., laboratory results, images). Primary care providers submitting the request for consult can book a phone consultation with a specific specialist of their choosing. The service is offered at no cost to patients and providers, and fee-for-service specialists and family physicians are reimbursed using existing provincial billing codes. The encounter consists of a brief phone call (typically about 10 min) between the providers that occurs on a date and time specified by each provider. At the conclusion of each phone consult, the specialist completes a consult report summarizing the advice given. The platform complies with all applicable Canadian healthcare privacy legislation including PIPEDA and provincial privacy acts (PHIA, PHIPA, HIA, etc.). It also secures patient data using Medstack which is a data security compliance platform for digital health applications that adheres to all standard healthcare security frameworks including: HIPAA, SOC2 and ISO 27001.

### Study design

This was a cross-sectional survey of all healthcare providers using the Virtual Hallway platform from July 14 to November 10, 2022 to determine the acceptability and feasibility of peer-to-peer phone consultations among healthcare providers.

A population-based sampling approach was used. This study was conducted in Nova Scotia, Canada between July 14, 2022 and November 10, 2022. This study conforms to the STROBE (Strengthening the Reporting of Observational Studies in Epidemiology) guidelines for reporting cross-sectional, observational studies.

The survey was developed using an iterative participatory design including the investigators and users of the platform to ensure it aligned with the needs of both the clinician users and the local health system (8). The questions were designed to assess the acceptability of the platform as well as the need for and quality of subsequent in-person referral. At the close of each consultation, the specialist and the requesting provider were requested to complete closeout surveys embedded within the Virtual Hallway platform related to the experience of the completed consult. The questions are found in supplement A.

### Recruitment and respondent characteristics

Participants were recruited using a convenience sample of the entire population of interest. All providers on the Virtual Hallway platform interested in conducting phone consults and licensed to practice medicine in the province of Nova Scotia were eligible to create an account in the system and participate in synchronous provider-to-provider virtual phone consults. Physicians, NPs, and specialists were informed of the service through a combination of email communications, fax communication, and general information available on the website. All providers that were already registered at the time of study initiation were able to complete the questionnaire each time they completed a phone advice call. There were a limited number of volunteer participants which mitigated potential risks from this research and data collection ensured participant privacy by de-identifying the information collected. An exemption letter was obtained from the Nova Scotia Health Research Ethics Board as a program evaluation study in accordance with Chapter 2 of the Tri-Council Policy Statement guidelines.

### Data collection and analysis

The digital questionnaire was administered through the platform at the end of each virtual consultation. Characteristics of respondents were described using appropriate univariable statistical approaches for continuous and categorical data. Likert scale responses to survey questions were reported as medians with interquartile ranges and the proportion responding to each category were also described.

Questions such as, level of satisfaction with consult experience were captured in a 5-point Likert-scale ranked from 1 (strongly disagree) to 5 (strongly agree), with 3 being neutral. These responses were analyzed by computing descriptive statistics (mean, median, mode and standard deviation). Mean Likert scale values were interpreted as the overall agreement toward a variable in the questionnaire. To determine the statistical significance of the difference of the mean from the neutral value, we performed the one-sample *t*-test (alpha level = 0.05).

To analyze dichotomous variable responses (yes/no), such as referral avoidance due to phone consultation, we measured the frequency distribution of the overall responses and by specialty groups to determine response variance over different specialties.

## Results

### Response rate and respondent characteristics

Two-hundred and eleven PCPs from across Nova Scotia participated in the pilot study by completing at least one or more consults along with the associated closeout survey(s). This accounted for approximately 15% (211/1357) of the active PCPs in the province. The survey response rate by PCPs was 81.8%, whereas for specialists the response rate was 72.1%. We received 654 closeout survey responses from specialists; for data completeness we excluded incomplete responses, leaving a total 632 responses. There were 34 specialists who participated across 17 specialty areas (mean number of cases per provider = 17.7, sd 27.3 range 2–156). We received 614 closeout survey responses from PCPs. There were 608 after excluding incomplete responses. A total of 181 PCPs participated (mean number of cases per provider = 3.33, sd 4.8, range 1–45).

**Figure 1:**
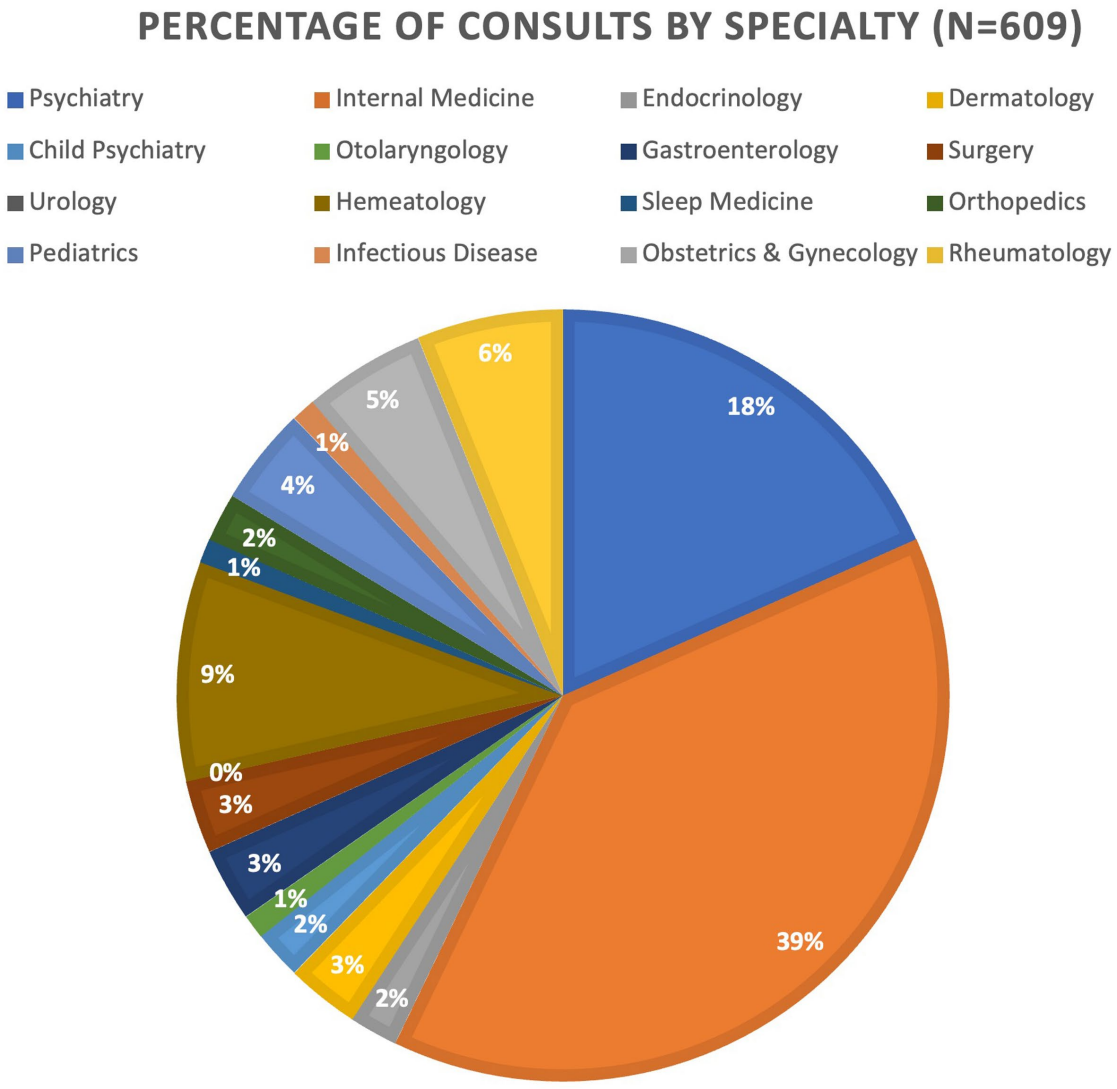
Percentage of total consults by specialty during pilot study.

The most consulted specialties were general internal medicine (39%) and psychiatry (18%), followed by rheumatology (6%) and obstetrics and gynecology (5%) (Figure 1).

### Impact on need for formal consultation

PCP survey results indicated that 84% (511/608) of phone consults resulted in avoidance of the need for an in-person referral (Table 1). Among the remaining 16% of phone consults that did not eliminate the need for a referral, the PCP respondents indicated that none (0%) of those were initially intended to avoid an in-person referral. PCPs indicated that for 87% of the phone advice calls that ended up requiring a formal consultation, that the phone advice improved the quality of the in-person referral. Ninety percent of PCP consultations were found to have enhanced the patient’s care while they awaited an in-person appointment with a specialist.

**Table 1:** Result of the closeout survey of referring physicians and nurse practitioners on closing the phone consult.

Did this Virtual Hallway consultation avoid the need for an in-person referral?	
	Cases reviewed between [2022-07-14] and [2022-11-22] *n* = 608
Yes	84%
No	16%
Subset of survey to those referring physicians and practitioners that answered No. (*n* = 97)	
Was this consultation intended to avoid referral?	
Yes	0%
No	100%
Did this consult improve the quality of your referral?	
Yes	87%
No	13%
Did this consultation improve the patient’s care while they wait for an in-person referral?	
Yes	90%
No	10%

Stratified analysis by specialty revealed variability in the percentage of cases avoiding a formal in-person consultation. PCPs reported the avoidance of formal in-person specialist consultation in 93.7% (194/207) of phone advice calls with internal medicine followed by 87.8% (101/115) with psychiatry—with at least 40% of in-person referrals being avoided across all specialties. All responses by PCPs on avoiding a formal in-person consultation by specialty is provided in Table 2.

**Table 2:** Result of the closeout survey of PCP consultants on closing the phone consult by specialty of the consultation.

Did this Virtual Hallway consultation avoid the need for an in-person referral?		
	Total consults	Answer yes: *n* (%)
Internal Medicine	207	194 (93.72%)
Psychiatry	115	101 (87.83%)
Infectious Disease	8	7 (87.5%)
Endocrinology	42	36 (85.71%)
Rheumatology	34	27 (79.41%)
Hematology	43	34 (79.07%)
Pediatrics	19	15 (78.95%)
Dermatology	18	13 (72.22%)
Obstetrics and Gynecology	37	26 (70.27%)
General Surgery	13	9 (69.23%)
Gastroenterology	19	13 (68.42%)
Child and Adolescent Psychiatry	12	8 (66.67%)
Orthopedics	12	8 (66.67%)
Otolaryngology	8	5 (62.5%)
Urology	6	3 (50%)
Pain Medicine	5	2 (40%)

A total of 608 responses were received from specialists for the post-phone consult survey (Table 3). Specialists reported that 42% of the cases reviewed would have been at least somewhat necessary for an in-person consultation had the referral been made through traditional in-person routes. In contrast, in-person consultations were deemed unnecessary for 58% of the cases.

**Table 3:** Result of the closeout survey of specialist consultants on closing the phone consult.

If this patient had been referred directly to your clinic, would the referral have been:	
Answer	Cases reviewed between [2022-07-14] and [2022-11-10] (*n* = 608)
Necessary	26%
Somewhat necessary	16%
Somewhat unnecessary	10%
Unnecessary	48%

### User satisfaction

**Figure 2:**
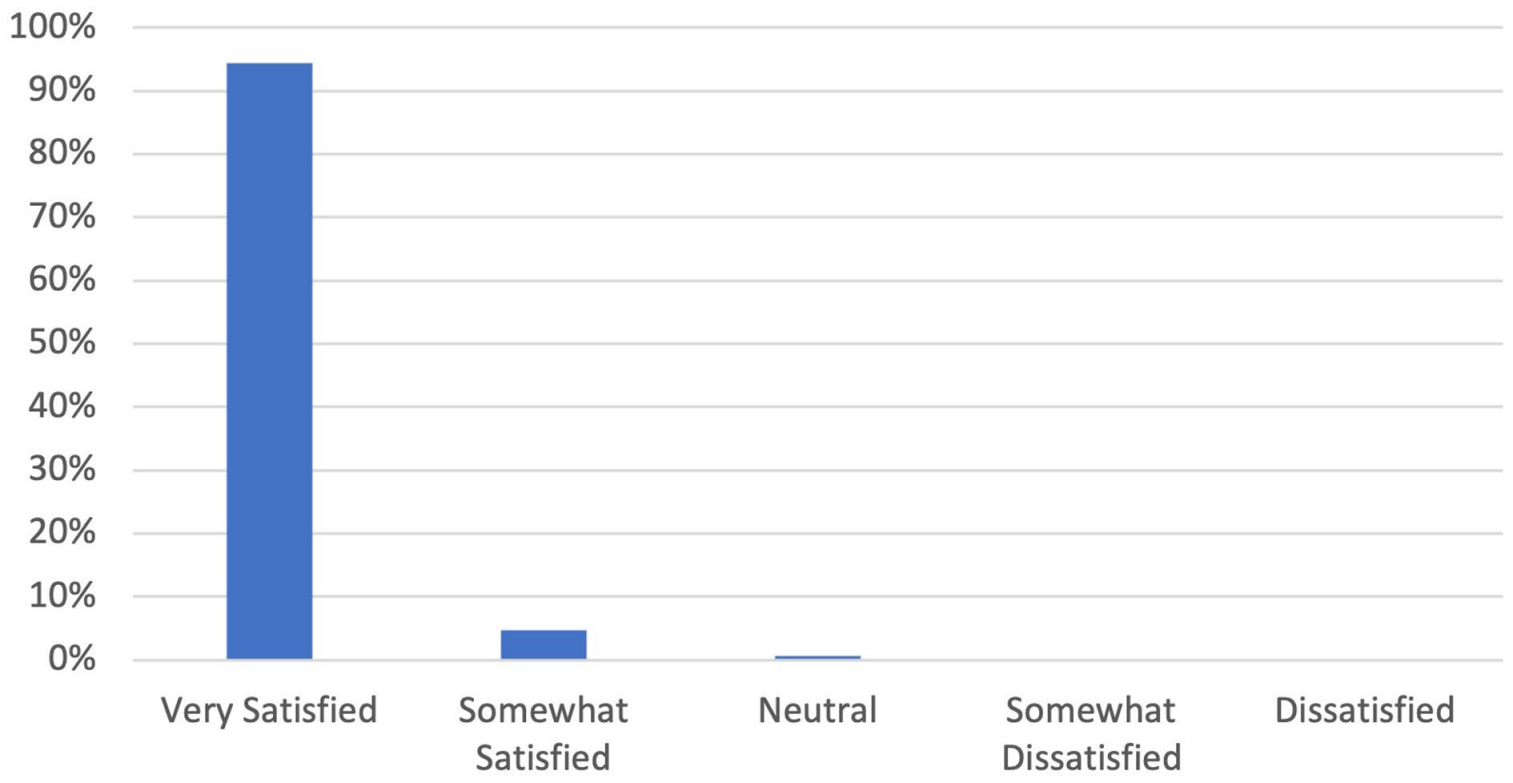
PCP satisfaction survey.

**Figure 3:**
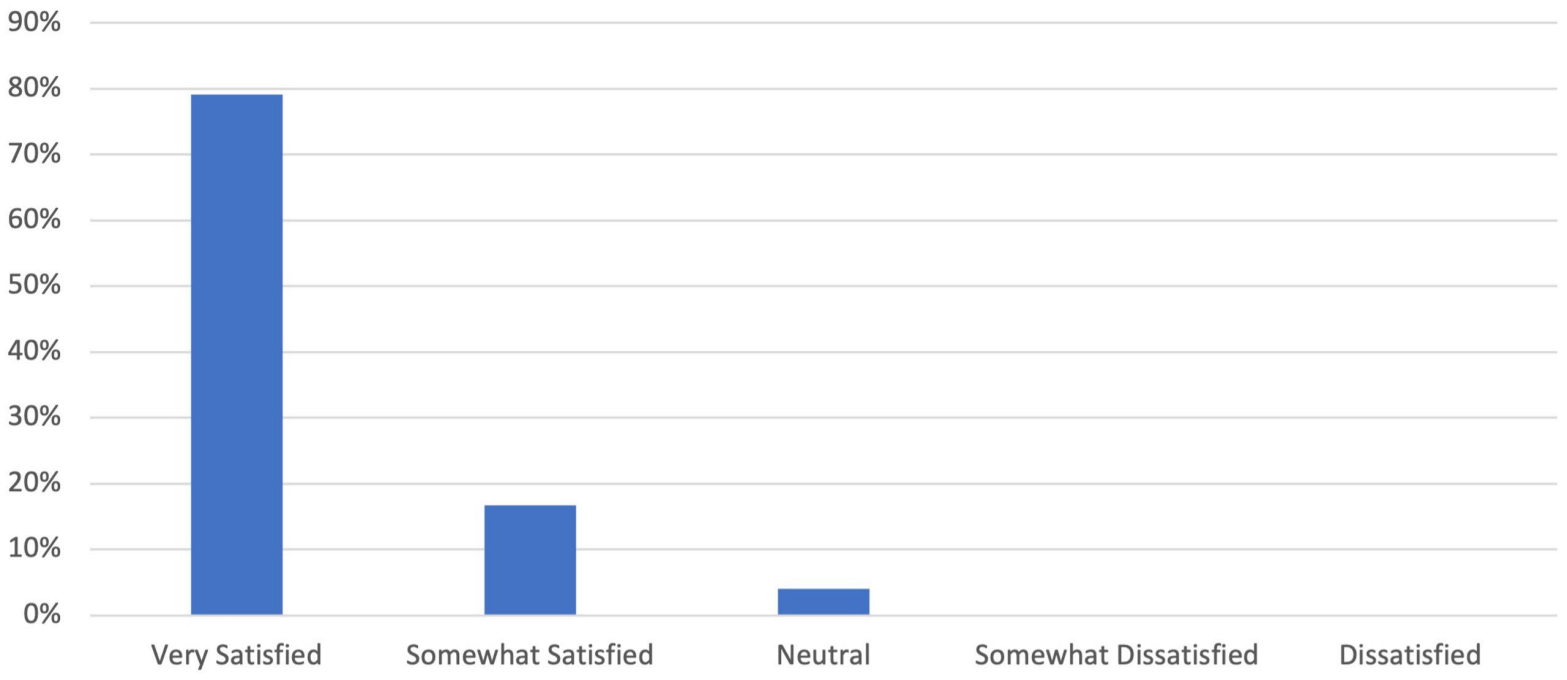
Specialist survey.

Nearly all PCPs reported a high level of satisfaction with their consult experience, with 99% indicating they were either “very satisfied” or “satisfied” (Figure 2) (mean 4.93, sd 0.29, *p* < 0.01). Ninety-six percent of specialists were either “very satisfied” or “satisfied” with the consult experience (Figure 3) (mean 4.75, sd 0.54, *p* < 0.01).

### Billing volumes

**Figure 4:**
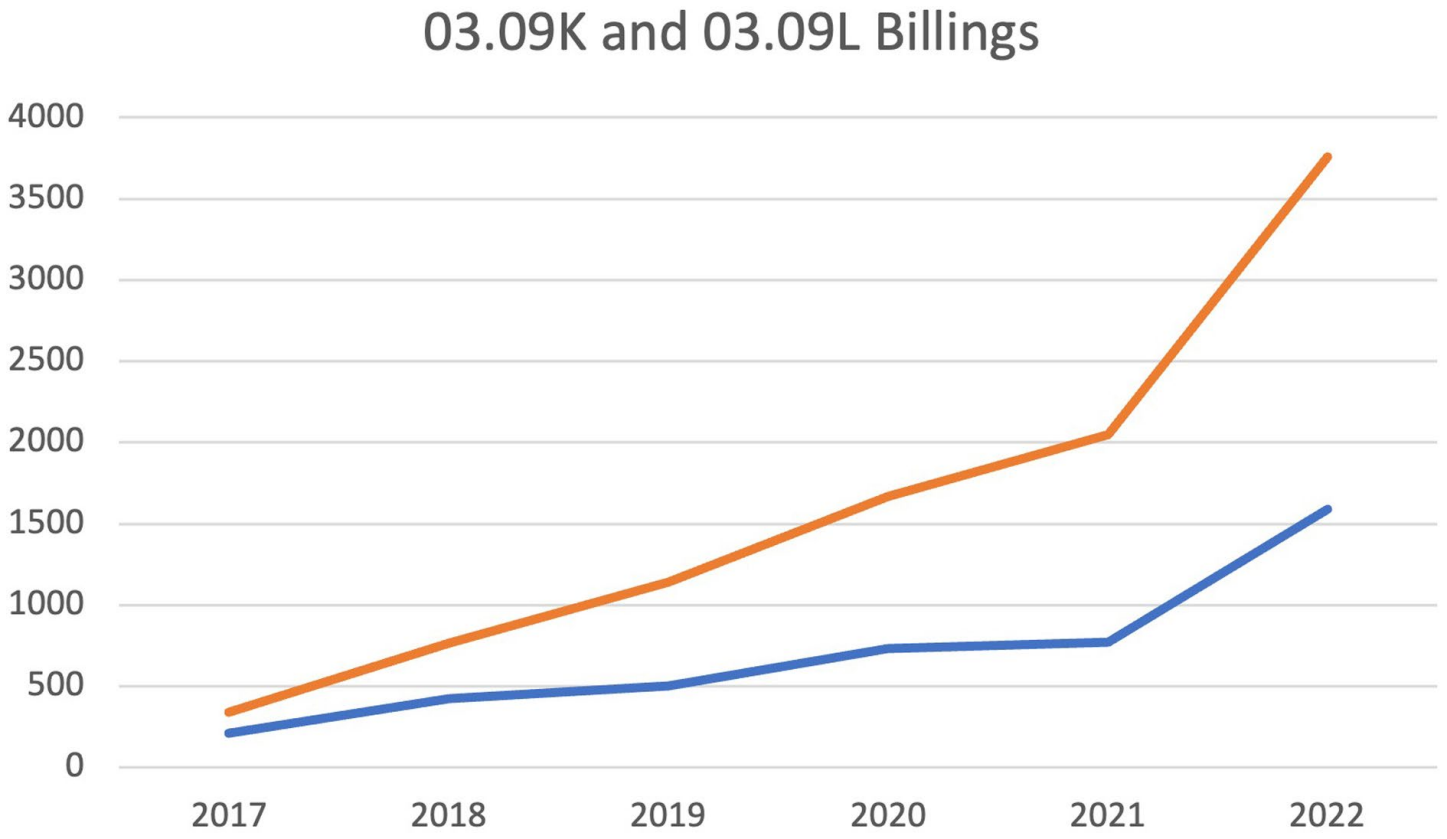
Total number of Nova Scotia province-wide 03.09 K and 03.09 L billing submissions by year. Deflection point reflects timepoint when Virtual Hallway was initiated for this study.

The number of synchronous provider-to-provider virtual consultations are reflected in billing volumes for codes 03.09 K (billed by specialist provider completing consultation) and 03.09 L (billed by referring provider requesting consultation). Although volumes have increased progressively since the introduction of these billing codes in 2017, there is a notable deflection in volumes from 2021 to 2022 which correlates to the time period of this study (Figure 4).

## Discussion

This study examined the acceptability and impact of synchronous provider-to-provider communication for improving specialty care access within the Canadian healthcare landscape using a novel digital platform. The aims of the study were to determine to what extent synchronous provider-to-provider consultations reduce the need for in-person specialist appointments, determine the acceptability of the platform, and to describe utilization patterns of these consults by healthcare providers.

Our findings demonstrate a higher-than-average physician response rate and that phone consultations significantly reduce the need for in-person referrals—with higher referral avoidance compared to other modalities of peer-to-peer communication found in the literature (e.g., eConsults). We also found that even those consults that go on to in-person referrals are enhanced through phone consultation. Of note, there was discrepancy found between PCPs and specialists regarding perceived need for in-person referrals following phone consults, although there were uniformly high satisfaction ratings for these phone interactions.

The primary finding is that PCPs identified that 84% of all synchronous provider-to-provider consultations avoided the need for an in-person referral. In Canada, wait times for specialist appointments are an all-time high (1). As a result, these synchronous provider-to-provider consultations could have significant impacts on alleviating the burden on waitlists and improving access to specialty care. If this finding continues to be replicated, then this model of consultation offers potential time and cost advantages compared to in-person referrals. Consequently, synchronous provider-to-provider consultations may provide a more efficient and affordable approach to specialty care access, reducing the burden on healthcare systems and patients alike (7).

In addition to referral avoidance, this study revealed that in cases where the synchronous consultations did not eliminate the need for an in-person referral, they provided valuable benefits through an improvement in the quality of the subsequent in-person referral or an optimization of patient management while on the waitlist. Interestingly, our study identified a discrepancy between the perceptions of specialists and PCPs regarding the necessity of referrals. Specialists indicated that over half of the phone consults (58%) would have been unnecessary or somewhat unnecessary if they had been referred in-person, whereas PCPs reported that 84% of cases would have been referred for in-person consultations if not for the phone consult. This incongruence highlights the need for improved communication and understanding between specialists and PCPs to optimize the referral process and resource allocation in the healthcare system (9).

The potential to reduce waitlists is a significant advantage of synchronous provider-to-provider consultations. Our findings are consistent with previous research on electronic consultations (eConsults), which have been shown to improve access to specialty care and reduce wait times; with up to 65% of eConsults avoiding the need for in-person specialist referral (6, 10, 11). The current study found potential referral avoidance beyond the upper range of these studies. One possibility for the disparity between these two peer-to-peer consultation methods is that phone consults allow a synchronous dynamic conversation to take place, being able to clarify and ask questions, which may allow a greater scope of consultation. As described from one of the physician focus groups in the Cook et al. (12) study: “I find the value of communicating on the phone because it’s two ways, back and forth, and then I get my answers right away” (12).

By decreasing the number of unnecessary in-person referrals, phone consultations could contribute to more efficient resource allocation and streamlined access to specialty care for patients in need.

The satisfaction ratings reported among healthcare providers suggests a high level of acceptability of this model of synchronous provider-to-provider consultations. Provincial billing volumes demonstrated a progressive increase in uptake of provider-to-provider synchronous consultation since compensation was first introduced in 2017. There is a notable deflection point in the volume data from 2021 to 2022, which correlates to the significant expansion of the platform users within the province. The platform supported increased uptake of phone consultation relative to the status quo of unsupported booking, documentation, and billing. Although compensation for this form of care is available independent of the Virtual Hallway platform, there are significant administrative barriers related to booking times when both referring, and specialist, physicians are available. This is one of the major advantages of the platform, as it facilitates physician-to-physician communication without administrative staff, or the complex scheduling previously required among clinics. Additional benefits of the platform include ease of documentation and automated billing functions, all of which minimize the administrative burden associated with care delivery.

One of the strengths of this study was having a high response rate (72% for specialists and 82% for PCPs) given the typically low historical response rates for physician surveys (typically well below 50%) (13, 14). This may indicate that this system, with immediate, real-time feedback, provides a unique and effective way of surveying physicians compared to alternative strategies such as email reminders, financial incentives or even personalization, all of which have uncertain benefits and significant variability in the literature (14). The high satisfaction ratings by both PCPs and specialists also suggests high feasibility for implementation into clinical practice across primary care and specialty areas.

Limitations of our study include the use of a convenience sample and a focus on a single Canadian province, which may limit the generalizability of the results. Additionally, the study did not assess the long-term impact of phone consultations on patient outcomes or healthcare system performance. Another limitation includes the survey questions which may introduce potential bias toward positive responses. Furthermore, this was a pilot study and may not have had a sample size sufficient to detect a true effect and may be subject to selection bias. Results should not be interpreted as providing conclusive evidence, but rather as guidance for future research. Future research should utilize objective measures of healthcare quality, outcome, and utilization to evaluate the effectiveness of phone consultations in other healthcare settings and populations. Comparative studies between phone consults and other forms of peer-to-peer communication should be conducted to understand the relative benefits of different modalities.

In conclusion, this pilot study demonstrates the potential of provider-to-provider synchronous virtual care to address the pressing issues of waitlists and access to specialty care in the Canadian healthcare system and the benefit of a novel digital platform in supporting uptake of this care modality. The findings suggest that phone consultations are well-accepted among healthcare providers and can avoid a significant proportion of in-person referrals. Moreover, phone consultations may provide a more time-efficient and cost-effective alternative to in-person referrals, with the potential to reduce waitlists, improve patient outcomes, and enhance the overall quality of care.

## Data availability statement

The raw data supporting the conclusions of this article will be made available by the authors, without undue reservation.

## Ethics statement

The studies involving humans were approved by Nova Scotia Health Research Ethics Board. The studies were conducted in accordance with the local legislation and institutional requirements. The ethics committee/institutional review board waived the requirement of written informed consent for participation from the participants or the participants’ legal guardians/next of kin because research relies exclusively on secondary use of anonymous information, with the process of data recording and dissemination of results not generating identifiable information.

## Author contributions

KT: Data curation, Formal analysis, Writing – original draft, Writing – review & editing. JJ: Conceptualization, Investigation, Methodology, Writing – original draft, Writing – review & editing. AM: Formal analysis, Writing – review & editing. AA: Data curation, Formal analysis, Methodology, Writing – original draft, Writing – review & editing. SM: Validation, Writing – review & editing. DR: Conceptualization, Methodology, Writing – original draft, Writing – review & editing. JC: Conceptualization, Methodology, Writing – original draft, Writing – review & editing.

## Footnotes

^1^ https://msi.medavie.bluecross.ca/wp-content/uploads/sites/3/2023/08/Interim-Fee-Reference-Guide.pdf
